# Association of conventional cigarette smoking, heated tobacco product use and dual use with hypertension

**DOI:** 10.1093/ije/dyae114

**Published:** 2024-08-21

**Authors:** Huan Hu, Tohru Nakagawa, Toru Honda, Shuichiro Yamamoto, Tetsuya Mizoue

**Affiliations:** Research Center for Prevention from Radiation Hazards of Workers, National Institute of Occupational Safety and Health, Kanagawa, Japan; Department of Epidemiology and Prevention, Center for Clinical Sciences, National Center for Global Health and Medicine, Tokyo, Japan; Hitachi Health Care Center, Hitachi, Ltd, Ibaraki, Japan; Hitachi Health Care Center, Hitachi, Ltd, Ibaraki, Japan; Hitachi Health Care Center, Hitachi, Ltd, Ibaraki, Japan; Department of Epidemiology and Prevention, Center for Clinical Sciences, National Center for Global Health and Medicine, Tokyo, Japan

**Keywords:** Heated tobacco products, cigarettes, smoking, hypertension

## Abstract

**Background:**

Heated tobacco products (HTPs) have emerged as alternatives to conventional cigarettes. However, their health effects remain largely unknown. This study aimed to prospectively explore the association between the use of cigarettes and HTPs and the risk of hypertension.

**Methods:**

This cohort study analysed data from 30 152 workers (82.0% men, mean age 42.9 ± 11.0 years) who were initially free of hypertension, participating in the Japan Epidemiology Collaboration on Occupational Health Study. Participants were categorized into five groups based on their self-reported tobacco product use: never smokers, past smokers, exclusive cigarette smokers, exclusive HTP users and dual users of cigarettes and HTPs. Hypertension cases were identified using three data points from annual health checkup data collected between 2019 and 2021. Cox proportional hazards regression models were used to investigate the association between tobacco product use and hypertension.

**Results:**

During a mean follow-up of 2.6 years (range: 0.1–4.0 years), 3656 new cases of hypertension were identified. Compared with never smokers, the risk of hypertension was higher among exclusive cigarette smokers [hazard ratio (HR) 1.26, 95% confidence interval (CI) 1.13–1.41] and exclusive HTP users (HR 1.19, 95% CI 1.06–1.34). There was also a suggestion of increased risk of hypertension among dual users (HR 1.16, 95% CI 0.98–1.38). Furthermore, the risk of hypertension increased with the intensity of cigarette/HTP use in all tobacco product users.

**Conclusions:**

Similarly, both cigarette smoking and HTP use elevate the risk of hypertension. HTPs should not be regarded as less harmful alternatives to traditional cigarettes for preventing hypertension.

Key Messages

- This is the first large prospective study to investigate whether the use of heated tobacco products (HTPs) is associated with an increased risk of hypertension.
- We found that both exclusive HTP use and dual use of HTPs and cigarettes are associated with an elevated risk of hypertension.
- These findings suggest that HTPs may not be a safer alternative to conventional cigarettes regarding hypertension risk.

## Introduction

Cigarette smoking is a major risk factor for various diseases, including cancer, heart disease and other chronic diseases.^1^ Globally, the prevalence of smoking has declined over the past decades.^2^ Since Philip Morris International (PMI) launched its IQOS line of heated tobacco products (HTPs) and electronic cigarettes in 2014, HTPs have been marketed in over 70 countries.^3^^,^^4^ PMI is now introducing more affordable versions to expand their reach, potentially targeting vulnerable populations like adolescents and those in low and middle-income countries.^4^^,^^5^ Japan has emerged as one of the largest markets for HTPs, where nearly half of the current tobacco product users report using HTPs exclusively or concurrently with other tobacco products.^6^ HTPs heat tobacco within a specific temperature range, generating aerosols containing harmful or potentially harmful compounds similar to those in cigarette smoke, albeit at lower concentrations.^7^ Epidemiological evidence regarding the health risks associated with using these products is scarce.

Hypertension is the leading cause of premature cardiovascular death, affecting over one billion people globally.^8^ Cigarette smoke contains toxicants such as nicotine and heavy metals, which have been shown to increase blood pressure in animal experiments.^9–11^ Although conventional cigarette smoking causes a temporary rise in blood pressure,^12^ evidence of the association between cigarette smoking and hypertension is conflicting; some cohort studies have reported a positive association,^13–17^ whereas others have not.^18^^,^^19^ Notably, HTP aerosols contain comparable levels of nicotine,^7^ and HTP use has been found to induce endothelial dysfunction,^20^ raising concerns about the potential adverse effects of HTPs on hypertension. Clinical trials have demonstrated that HTP use can lead to temporary and acute increases in blood pressure.^21^^,^^22^ However, the long-term association between HTP use and the risk of hypertension remains unknown. Additionally, the dual use of cigarettes and HTPs further complicates the potential relationship between tobacco use and hypertension.

To address these knowledge gaps, this cohort study aimed to investigate the association between cigarette smoking, HTP use, dual use of cigarettes and HTPs and the risk of hypertension among the Japanese working population, with annual data collection on smoking status during the follow-up period.

## Methods

### Study setting

The Japan Epidemiology Collaboration on Occupational Health (J-ECOH) Study is an ongoing study involving workers from more than ten companies across various industries, including electric machinery, steel, chemical, automobile, plastics manufacturing and healthcare. We invited companies headquartered in the Kanto and Tokai regions of Japan, via an occupational physician network (convenience sampling). Data were collected annually from each participating company through company-organized health checkups, which included anthropometric measurements, physical exams, laboratory tests and self-administered questionnaires on medical history and lifestyle. Further details have been reported elsewhere.^23^

The present sub-study used data from a large J-ECOH Study participating company, an electrical machinery manufacturer, which has been collecting annual data on new tobacco products, including HTPs, since 2018. The company, with ∼50 000 workers in 2018, implemented measures against passive smoking in indoor workplaces by either maintaining a completely smoke-free environment or providing spatially separated smoking areas, depending on the location.

### Analytic cohort

We created an analytic cohort of health checkup examinees during the 2018 fiscal year (baseline) and followed them until May 2022, based on annual checkups. Of 49 731 examinees ≥20 years, we excluded individuals with hypertension (receiving antihypertensive treatment or blood pressure ≥140/90 mmHg) (*n* = 9093), those with missing information on either baseline hypertension status (*n* = 451) or smoking status (*n* = 6892), and individuals with a history of cancer or cardiovascular disease (*n* = 629) at baseline. Of the remaining 32 666 participants, we further excluded those who did not attend any subsequent health checkups (*n* = 2480) or who attended but did not undergo blood pressure measurement (*n* = 34), resulting in a final sample size of 30 152 participants. Workers included in the final sample were younger and had lower rates of diabetes and dyslipidaemia, as well as lower blood pressure levels compared with those excluded. Only minor differences were observed in terms of body mass index (BMI), tobacco product use and other health behaviours (Supplementary Table S1, available as Supplementary data at *IJE* online).

### Exposure

Tobacco product use was ascertained using a self-administered questionnaire as part of a health checkup, with smoking-related questions detailed in Supplementary Table S2, available as Supplementary data at *IJE* online. Examinees were queried about their current use of any tobacco products, including conventional cigarettes, e-cigarettes and HTPs. The response options were ‘Never’, ‘Quit’, and ‘Yes, current use’, with ‘current use’ including both daily and non-daily use. Those who reported current use of tobacco products were further asked about the types of tobacco products they used, with responses of ‘Conventional cigarettes only’, ‘E-cigarettes/HTPs only’, or ‘Both (cigarettes and e-cigarettes/HTPs)’ In Japan, e-cigarettes without nicotine are not regulated or classified as tobacco products by law. Nicotine-containing e-cigarettes are regulated as medicinal products and cannot be legally sold, though they can be imported for personal use. It has been reported that only about 1% of people in Japan use e-cigarettes,^24^^,^^25^ and our previous study showed that over 95% of new tobacco product users reported using HTPs.^26^ Since the questionnaire did not distinguish between HTPs and e-cigarettes, we assumed them to be HTPs. Based on the participants’ responses, the individuals were categorized into five groups: never smokers, former smokers, exclusive cigarette smokers, exclusive HTP users and dual users of cigarettes and HTPs.

The questionnaire also included a question about the average number of cigarettes/HTPs used per day. For dual users, this quantity represents a combined total of both products. To investigate the association between the intensity of cigarette/HTP use and the risk of hypertension, exclusive cigarette smokers, exclusive HTP users and dual users were further categorized into two groups based on their daily consumption: 1–10 and ≥11 cigarettes/HTPs.

### Outcome

The annual health checkup was conducted at the company’s healthcare centre, local clinics or onsite in meeting rooms, depending on the worksite location. Blood pressure was measured using an automatic blood pressure monitor. Prior to the measurement, participants were instructed to rest in a chair for ∼5 min. For individuals with an initial systolic blood pressure measurement ≥140 mmHg and/or diastolic blood pressure ≥90 mmHg, blood pressure was measured twice, 2–3 min apart and the lower measurement was used. The onset of hypertension was determined using annual health checkup data collected from baseline until the 2021 fiscal year. Hypertension was defined as meeting at least one of the following criteria: a systolic blood pressure level of ≥140 mmHg, a diastolic blood pressure level of ≥90 mmHg or receiving antihypertensive treatment.^27^

### Covariates

Informed by previous research and guided by a Directed Acyclic Graph (Supplementary Figure S1, available as Supplementary data at *IJE* online), the following covariates were considered: age, sex, job position (as an indicator of socioeconomic status),^28^ sleep duration,^29^ leisure-time physical activity,^30^ alcohol consumption,^31^ high-sodium food consumption,^32^ BMI,^33^ dyslipidaemia,^34^ diabetes,^35^ and baseline systolic blood pressure. During our analysis, we observed high correlations (*r* ≈ 0.60) among dietary factors, including high-sodium food, vegetable, fruit and dairy consumption (dietary-related questions detailed in Supplementary Table S2, available as Supplementary data at *IJE* online). To avoid multicollinearity, we exclusively included high-sodium food consumption in our model due to its causal effect on hypertension.

Body weight and height were measured using a scale with the participants wearing light clothes and no shoes. BMI was calculated as weight in kilograms divided by height squared in meters. Job position (high rank or others), sleep duration (<5, 5 to <6, 6 to <7, ≥7 h/day), alcohol consumption [non-drinkers, drinkers consuming <1, 1 to <2 or ≥2 *go*/day; *go* is a traditional Japanese unit of volume and one *go* (180 mL) of sake contains ∼23 g of ethanol] and leisure-time physical activity (≥150 min/week or <150 min/week) were ascertained via a self-administered questionnaire. Additionally, participants were asked about the frequency of high-sodium food consumption, ranging from ‘rarely’ to ‘≥3 times per day’. We transformed this into a continuous measure (times per week) by assigning numeric values to each category, from 0 for ‘rarely’ to 21 times per week for ‘3 times per day’.

Dyslipidaemia was defined as a triglyceride level ≥150 mg/dL, low-density lipoprotein cholesterol level ≥140 mg/dL, high-density lipoprotein cholesterol level <40 mg/dL or receiving medical treatment for dyslipidaemia. Diabetes was defined as a fasting blood glucose ≥126 mg/dL, a HbA1c level ≥6.5% or receiving medical treatment for diabetes.

### Statistical analysis

The baseline characteristics of the study participants were described as means for continuous variables and percentages for categorical variables. Chi-squared tests for categorical variables and analysis of variance for continuous variables were used to examine differences in baseline characteristics among different tobacco use groups. Time to event or time to censoring was calculated as the time between the baseline health checkup and the earliest of the following dates: (i) the estimated onset date of hypertension, determined as the midpoint between the checkup when hypertension was first detected and the previous checkup, (ii) the date of the checkup when a participant first reported a change in smoking status or (iii) the date of the last health checkup attended. We censored participants whose smoking status changed from baseline, excluding subsequent exposures such as switching from a former smoker at baseline to a current smoker during follow-up, due to their short duration in the study.

The Cox proportional hazards regression was used to estimate hazard ratios (HRs) and 95% confidence intervals (CIs) for the association between HTP use and hypertension. We assessed the proportional hazards assumption using Schoenfeld residuals and found no violations. In the first model, age, sex and job position were adjusted. In the second model, we further adjusted for health behaviours such as sleep duration, leisure-time physical activity, alcohol consumption and high-sodium food consumption. In the third model, we expanded the adjustments to include BMI, dyslipidaemia, diabetes and baseline systolic blood pressure. We performed multiple imputations by fully conditional specification to address missing data on covariates at baseline, with missingness ranging from 0% to 18%, and smoking status during follow-up visits (Supplementary Figure S2, available as Supplementary data at *IJE* online). This involved generating 15 imputed datasets, analysing each dataset with the respective model and combining the results using Rubin’s rule to obtain a final point estimate and standard error.

In the sensitivity analysis, we used the last observation carried forward method to impute missing smoking data at follow-ups, assessing the robustness of the main results. All the statistical analyses were performed using SAS version 9.4 (SAS Institute, Cary, NC, USA).

## Results

Of 30 152 participants included in the analysis, 82.0% were men and the mean (SD) age at baseline was 42.9 (11.0) years. The prevalence of exclusive cigarette smoking, exclusive HTP use and combined use was 12.3%, 9.4% and 5.2%, respectively. Table 1 presents the baseline characteristics of the study population according to tobacco product use. Notably, participants who exclusively used HTPs were younger than those who exclusively smoked cigarettes. Compared with never smokers, tobacco product users were older, more likely to be men, and had a higher prevalence of diabetes and dyslipidaemia. Mean blood pressure levels were similar across the groups.

**Table 1.** Baseline characteristics of study participants^a^

			Current use of tobacco-related products			
Characteristic	Never smoker	Past smoker	Exclusive cigarette smoker	Exclusive HTP user	Dual user	*P*
*N*	15 940	6078	3716	2837	1581	<0.001
Age, years, mean (SD)	40.2 (11.5)	47.1 (9.8)	46.4 (9.4)	43.9 (8.7)	43.8 (9.5)	<0.001
Male, *n* (%)	11 444 (71.8)	5600 (92.1)	3460 (93.1)	2709 (95.5)	1504 (95.1)	<0.001
Body mass index, kg/m^2^, mean (SD)	22.9 (3.5)	23.6 (3.1)	23.4 (3.6)	23.9 (3.5)	23.9 (3.7)	<0.001
Systolic blood pressure, mmHg, mean (SD)	117.0 (11.4)	119.4 (10.4)	117.4 (11.1)	118.4 (10.4)	117.7 (10.8)	<0.001
Diastolic blood pressure mmHg, mean (SD)	70.9 (9.0)	74.6 (8.3)	72.9 (8.8)	74.0 (8.4)	72.7 (8.70)	<0.001
Highly ranked job position, *n* (%)	2014 (13.4)	5855 (24.8)	583 (16.2)	512 (18.4)	245 (16.0)	<0.001
Alcohol consumption, *n* (%)	8381 (52.8)	4415 (72.8)	2421 (65.3)	1960 (69.2)	1064 (67.3)	<0.001
Short sleep duration, <5 h/day, *n* (%)	1583 (10.0)	532 (8.9)	400 (10.8)	242 (8.6)	198 (12.6)	<0.001
Leisure time physical activity, ≥150 min/week, *n* (%)	2157 (13.7)	1123 (18.8)	482 (13.0)	458 (16.2)	231 (14.7)	<0.001
High-sodium foods, times/week, mean (SD)	2.1 (2.3)	2.2 (2.4)	2.1 (2.4)	1.9 (2.2)	2.0 (2.3)	<0.001
Diabetes, *n* (%)	457 (3.7)	359 (6.3)	272 (7.6)	167 (6.1)	103 (6.8)	<0.001
Dyslipidaemia, *n* (%)	4741 (37.8)	2666 (46.5)	1819 (50.3)	1298 (47.3)	751 (49.4)	<0.001

HTP, heated tobacco product; SD, standard deviation.

a Data before multiple imputation were used.

As shown in Supplementary Figure S2, available as Supplementary data at *IJE* online, over 80% of participants attended all the subsequent health checkups between 2019 and 2021. The mean follow-up time for participants was 2.6 years (range: 0.1–4.0 years). A total of 3656 new-onset cases of hypertension were identified during 78 381 person-years of follow-up.

Table 2 shows adjusted HRs for hypertension according to tobacco product use. In the model adjusted for age, sex and job position (model 1), exclusive HTP users had a higher risk of hypertension (HR 1.26, 95% CI 1.13–1.42, *P *<* *0.001), followed by exclusive cigarette smokers (HR 1.20, 95% CI 1.08–1.34, *P *<* *0.001) and dual users (HR 1.21, 95% CI 1.02–1.43, *P *=* *0.03), compared with never smokers. This association persisted for exclusive HTP users (HR 1.17, 95% CI 1.04–1.32, *P *=* *0.008) after further adjusting for health behaviours (model 2), though somewhat attenuated. Even after adjusting for baseline BMI, diabetes, dyslipidaemia and systolic blood pressure (model 3), exclusive HTP users (HR 1.19, 95% CI 1.06–1.34, *P *=* *0.003) continued to have a higher risk of hypertension. An increased risk was also consistently observed among cigarette smokers in model 2 and model 3. Dual users (HR 1.16, 95% CI 0.98–1.38, *P *=* *0.09) had a marginally higher risk, likely due to the relatively small sample size.

**Table 2.** Prospective associations between tobacco product use and risk of hypertension

	Hazard ratio (95% CI)				
Model	Never smoker	Past smoker	Exclusive cigarette smoker	Exclusive HTP user	Dual user
Model 1^a^	1.00	1.13 (1.03–1.23)	1.20 (1.08–1.34)	1.26 (1.13–1.42)	1.21 (1.02–1.43)
Model 2^b^	1.00	1.05 (0.96–1.14)	1.12 (1.01–1.25)	1.17 (1.04–1.32)	1.12 (0.94–1.32)
Model 3^c^	1.00	1.01 (0.91–1.10)	1.26 (1.13–1.41)	1.19 (1.06–1.34)	1.16 (0.98–1.38)

HTP, heated tobacco product.

a Model 1 adjusted for age, sex and job position.

b Model 2 adjusted for age, sex, job position, short sleep duration, leisure-time physical activity, alcohol consumption and high-sodium food consumption.

c Model 3 adjusted for age, sex, job position, short sleep duration, leisure-time physical activity, alcohol consumption, high-sodium food consumption, body mass index, dyslipidaemia, diabetes and baseline systolic blood pressure.

Table 3 shows that higher intensity of tobacco product use is associated with an increased risk of hypertension among exclusive HTP users, exclusive cigarette smokers and dual users. Among exclusive HTP users, the adjusted HRs (95% CIs) in model 3 were 1.14 (0.93–1.41) for people who used 1–10 HTPs per day and 1.22 (1.06–1.40) for those who used ≥11 HTPs per day (*P* for trend = 0.003), compared with never smokers. Among exclusive cigarette smokers, the adjusted HRs were 1.24 (1.03–1.49) for 1–10 cigarettes per day and 1.27 (1.12–1.43) for ≥11 cigarettes per day (*P* for trend < 0.001). For dual users, the adjusted HRs were 0.87 (0.58–1.31) for 1–10 cigarettes/HTPs per day and 1.24 (1.03–1.50) for ≥11 cigarettes/HTPs per day (*P* for trend = 0.04).

**Table 3.** Prospective associations between intensity of tobacco product use and risk of hypertension

	Hazard ratio (95% CI)		
Tobacco product use group	**Model 1** ^a^	**Model 2** ^b^	**Model 3** ^c^
Exclusive cigarette smokers			
Never smokers	Reference	Reference	Reference
Exclusive cigarette smokers, 1–10 cigarettes/day	1.11 (0.93–1.33)	1.06 (0.89–1.27)	1.24 (1.03–1.49)
Exclusive cigarette smokers, ≥11 cigarettes/day	1.23 (1.09–1.39)	1.15 (1.02–1.30)	1.27 (1.12–1.43)
*P* for trend^d^	<0.001	0.02	<0.001
Exclusive HTP users			
Never smokers	Reference	Reference	Reference
Exclusive HTP users, 1–10 HTPs/day	1.16 (0.94–1.44)	1.12 (0.91–1.38)	1.14 (0.93–1.41)
Exclusive HTP users, ≥11 HTPs/day	1.31 (1.15–1.49)	1.23 (1.08–1.42)	1.22 (1.06–1.40)
*P* for trend^d^	<0.001	0.002	0.003
Dual users			
Never smokers	Reference	Reference	Reference
Dual users, 1–10 cigarettes and HTPs/day	0.89 (0.60–1.35)	0.86 (0.58–1.30)	0.87 (0.58–1.31)
Dual users, ≥11 cigarettes and HTPs/day	1.30 (1.08–1.57)	1.23 (1.02–1.48)	1.24 (1.03–1.50)
*P* for trend^d^	0.01	0.06	0.04

HTP, heated tobacco product.

a Model 1 adjusted for age, sex and job position.

b Model 2 adjusted for age, sex, job position, short sleep duration, leisure-time physical activity, alcohol consumption and high-sodium food consumption.

c Model 3 adjusted for age, sex, job position, short sleep duration, leisure-time physical activity, alcohol consumption, high-sodium food consumption, body mass index, dyslipidaemia, diabetes and baseline systolic blood pressure.

d Calculated using the median of each category as a continuous variable.

Sensitivity analyses found that the primary results did not change when using the last observation carried forward method to handle missing smoking data. The results are provided in Supplementary Tables S3 and S4, available as Supplementary data at *IJE* online.

## Discussion

This large prospective cohort study found that the use of HTPs, whether alone or in combination with cigarettes, was associated with an increased risk of hypertension in a dose-response manner. To the best of our knowledge, this is the first study to provide evidence of an association between HTP use and the risk of hypertension.

Our finding of a higher risk of hypertension among exclusive cigarette smokers aligns with existing cohort studies.^13–17^ For example, a USA-based cohort study (*n* = 17 539; mean age = 39.0 years) reported an increased risk of self-reported hypertension with exclusive cigarette smoking (adjusted HR = 1.21, 95% CI 1.06–1.38). However, some studies have shown mixed results, with some reporting no significant association (*n* = 4549; age = 45–74 years) and others noting differing effects by sex.^18^^,^^19^^,^^36^ Despite these discrepancies, our study supports a positive association between cigarette smoking and hypertension risk, after adjusting for potential confounding factors.

Although a common belief among cigarette smokers is that HTPs are less harmful than conventional cigarettes,^37^ our study revealed a notable connection between exclusive HTP use and an elevated risk of hypertension, which is in line with the results of clinical trials investigating the acute effect of HTP use on blood pressure.^21^^,^^22^ More importantly, we found a comparable increased risk of hypertension among exclusive HTP users and exclusive cigarette smokers, indicating that HTPs may pose a health risk comparable to traditional cigarettes in relation to hypertension. The market for HTPs has grown significantly in recent years, and these products are now available in over 70 countries.^3^^,^^4^ Our study provides important and timely evidence that HTP use may increase the risk of hypertension.

In our study, dual users of HTPs and cigarettes exhibited an increased risk of hypertension, particularly among those who consumed 11 or more cigarettes and/or HTPs per day. Notably, the daily consumption of tobacco sticks among dual users (16.8 ± 8.5) was similar to that of exclusive cigarette smokers (15.5 ± 6.4). This suggests that while dual users may smoke fewer conventional cigarettes, their overall tobacco consumption does not decrease. This finding aligns partially with a cross-sectional study from Hong Kong,^38^ which reported lower cigarette consumption but higher total tobacco consumption among dual users. Thus, dual use does not reduce total tobacco intake and may contribute to the increased risk of hypertension observed in this group.

Our findings suggest that HTPs may not be a safer alternative to conventional cigarettes regarding hypertension risk, which accounts for approximately half of cardiovascular disease events worldwide.^39^ These findings align with the recent US FDA report, which concluded that PMI’s current data do not demonstrate that IQOS use will significantly decrease the risk of tobacco-induced diseases for individuals or reduce harm to the population.^40^ The current study provides evidence that policymakers should regulate HTPs as stringently as conventional cigarettes to mitigate the potential health effects of these tobacco products.

Several potential mechanisms exist through which the use of tobacco products contributes to hypertension. One such mechanism involves the toxicants found in tobacco products, such as nicotine and heavy metals, which have been linked to elevated blood pressure.^7^^,^^9^^,^^10^^,^^11^^,^^41^ Specifically, nicotine inhalation has been found to affect pulmonary and systemic blood pressure in mice by promoting the activation of mitogen-activated protein kinase pathways and the renin–angiotensin–aldosterone system.^9^ The use of tobacco products causes various physiological changes, including sympathetic activation,^42^ oxidative stress,^43^ endothelial dysfunction,^20^^,^^44^ vascular injury,^45^ and increased arterial stiffness,^21^^,^^22^^,^^45^ all of which can contribute to hypertension.

## Strengths and limitations

Our study has several strengths, including a well-powered comparison of never, past and current smokers with annual smoking status data collection for censoring when smoking behaviour changed and comprehensive covariate adjustment. However, this study has several limitations. First, smoking habits were self-reported and not biologically verified (e.g. exhaled carbon monoxide or salivary cotinine). The resulting misclassification of smoking status might have led to an inaccurate assessment of the association. Second, in blood pressure measurements, random errors introduce the possibility of non-differential misclassification of hypertension, often resulting in a bias toward the null hypothesis. Third, residual and unmeasured confounding factors, such as second-hand exposure to cigarettes and HTPs and prior use of tobacco products before the baseline survey, may have influenced our results. Fourth, this study did not differentiate between daily and non-daily users, acknowledging that these groups may have different levels of health risk associated with their usage patterns. In addition, the questionnaire did not distinguish between HTPs and e-cigarettes, so we assumed them to be HTPs. As a result, e-cigarette users could be misclassified as HTP users, potentially underestimating the association between HTP use and hypertension. Finally, as our study was conducted within a single large company, the generalizability of our findings to populations with different backgrounds or from other industries may be limited.

## Conclusions

In this cohort study, cigarette smoking and HTP use were associated with an increased risk of hypertension. This suggests that HTPs should not be considered reduced-harm alternatives to traditional cigarettes for the prevention of hypertension.

## Ethics approval

Before data collection, we informed workers through posters about the J-ECOH Study and the use of their health data, owned by the company, for research purposes after anonymization. Workers could opt-out by notifying their occupational physicians, in compliance with Japanese Ethical Guidelines for observational studies. The study protocol including consent procedure was approved by the Ethics Committee of the National Center for Global Health and Medicine, Japan (NCGM-G-001140).

## Supplementary Material

dyae114_Supplementary_Data

## Data Availability

The data underlying this article cannot be shared publicly due to privacy/ethical reasons. The data will be shared on reasonable request to Dr Tetsuya Mizoue.
