# Supplementary material for: Association of conventional cigarette smoking, heated tobacco product use and dual use with hypertension
Source: Int J Epidemiol. 2024 Aug 21;53(5):dyae114. doi: 10.1093/ije/dyae114 (PMC11341126; doi:10.1093/ije/dyae114)
Supplement: dyae114_Supplementary_Data [file dyae114_supplementary_data.docx]

**Supplementary material**

**Supplementary Table S1 Characteristics of study participants and non-participants**

|  | Participants | Non-participants | *P* |
| --- | --- | --- | --- |
| N | 30 152 | 19 579 |  |
| Age, years, mean (SD) | 42.9±11.0 | 46.0±12.9 | <0.001 |
| Male, % | 82.0 | 84.2 | <0.001 |
| Body mass index, kg/m**^2^**, mean (SD) | 23.3±3.5 | 24.4±4.3 | <0.001 |
| Tobacco product use, % |  |  | <0.001 |
| Never smoker | 52.9 | 45.7 |  |
| Past smoker | 20.2 | 29.5 |  |
| Exclusive cigarette smoker | 12.3 | 12.8 |  |
| Exclusive HTP user | 9.4 | 7.4 |  |
| Dual user | 5.2 | 4.5 |  |
| High job position, % | 16.7 | 18.5 | <0.001 |
| Alcohol consumption, % | 60.7 | 55.2 | <0.001 |
| Short sleep duration (<5 hours/day, %) | 9.9 | 10.4 | 0.001 |
| Leisure time physical activity (≥150 minutes/week), % | 14.9 | 15.3 | 0.28 |
| High-sodium foods, times/week, mean (SD) | 2.1±2.3 | 2.2±2.5 | <0.001 |
| Systolic blood pressure, mmHg, mean (SD) | 117.7±11.1 | 126.2±15.2 | <0.001 |
| Diastolic blood pressure mmHg, mean (SD) | 72.2±8.9 | 78.3±12.1 | <0.001 |
| Diabetes, % | 5.3 | 15.3 | <0.001 |
| Dyslipidemia, % | 43.1 | 52.0 | <0.001 |

HTP, heated tobacco product; SD, standard deviation.

**Supplementary Table S2 Questions about smoking and dietary habits**

| 1 Are you currently using any tobacco products? (e.g., cigarettes, e-cigarettes, HTPs) | 1: Never  2: Quit  3: Yes |
| --- | --- |
| 2 For former and current users, how many do you use per day? | ( ) |
| 3 Which tobacco products are you using? | 1: Cigarettes only  2: E-cigarettes/HTPs only  3: Both (cigarettes and e-cigarettes / HTPs) |
| 4 In the past month, how often did you have the following items of food?  4.1. Raw vegetables, salad  4.2. Cooked vegetables (boiled and seasoned vegetables), dishes, or soups containing a lot of vegetables.  4.3. Fruit  4.4. Dairy  4.5. High sodium food (e.g., pickled plums, pickled vegetables, salted fish, salted fish eggs) | Options:  1: Rarely  2: 1-3 times per month  3: 1-2 times per week  4: 3-4 times per week  5: 5-6 times per week  6: Once a day  7: Twice a day  8: Three times a day |

e-cigarette, electronic cigarette; HTP, heated tobacco product.

**Supplementary Table S3 Prospective associations between tobacco product use and risk of hypertension (Missing smoking data imputed using last observation carried forward)**

|  | Hazard ratio (95% CI) | | | | |
| --- | --- | --- | --- | --- | --- |
|  | Never smoker | Past smoker | Exclusive cigarette smoker | Exclusive HTP user | Dual user |
| Model 1 | 1 | 1.13 (1.03, 1.23) | 1.20 (1.08, 1.34) | 1.26 (1.13, 1.42) | 1.22 (1.03, 1.44) |
| Model 2 | 1 | 1.05 (0.96, 1.14) | 1.12 (1.00, 1.24) | 1.17 (1.04, 1.32) | 1.12 (0.95, 1.33) |
| Model 3 | 1 | 1.01 (0.92, 1.10) | 1.26 (1.13, 1.40) | 1.19 (1.06, 1.34) | 1.17 (0.99, 1.39) |

Model 1 adjusted for age, sex, job position.

Model 2 adjusted for age, sex, job position, short sleep duration, leisure-time physical activity, alcohol consumption, high-sodium food consumption.

Model 3 adjusted for age, sex, job position, short sleep duration, leisure-time physical activity, alcohol consumption, high-sodium food consumption, body mass index, dyslipidemia, diabetes, and baseline systolic blood pressure.

HTP, heated tobacco product.

**Supplementary Table S4 Prospective associations between intensity of tobacco product use and risk of hypertension**

|  | Hazard ratio (95% CI) | | |
| --- | --- | --- | --- |
|  | Model 1 | Model 2 | Model 3 |
| Never smokers | Reference | Reference | Reference |
| Exclusive cigarette smokers, 1-10 cigarettes per day | 1.11 (0.92, 1.32) | 1.05 (0.88, 1.26) | 1.23 (1.03, 1.48) |
| Exclusive cigarette smokers, ≥11 cigarettes per day | 1.23 (1.09, 1.39) | 1.15 (1.02, 1.30) | 1.27 (1.12, 1.43) |
| *P* for trend^a^ | <0.001 | 0.03 | <0.001 |
|  |  |  |  |
| Never smokers | Reference | Reference | Reference |
| Exclusive HTP users, 1-10 HTPs per day | 1.18 (0.96, 1.45) | 1.13 (0.91, 1.39) | 1.15 (0.94, 1.42) |
| Exclusive HTP users, ≥11 HTPs per day | 1.31 (1.15, 1.49) | 1.23 (1.08, 1.41) | 1.21 (1.06, 1.39) |
| *P* for trend^a^ | <0.001 | 0.002 | 0.003 |
|  |  |  |  |
| Never smokers | Reference | Reference | Reference |
| Dual users, 1-10 cigarettes / HTPs per day | 0.88 (0.59, 1.31) | 0.85 (0.56, 1.27) | 0.86 (0.57, 1.28) |
| Dual users, ≥11 cigarettes / HTPs per day | 1.32 (1.10, 1.59) | 1.25 (1.03, 1.50) | 1.27 (1.05, 1.53) |
| *P* for trend^a^ | 0.006 | 0.04 | 0.03 |

Model 1 adjusted for age, sex, job position.

Model 2 adjusted for age, sex, job position, short sleep duration, leisure-time physical activity, alcohol consumption, high-sodium food consumption.

Model 3 adjusted for age, sex, job position, short sleep duration, leisure-time physical activity, alcohol consumption, high-sodium food consumption, body mass index, dyslipidemia, diabetes, and baseline systolic blood pressure.

a Calculated using the median of each category as a continuous variable.

HTP, heated tobacco product.

**Supplementary Figure S1 Directed acyclic graph illustrating the confounding effect of smoking on hypertension**


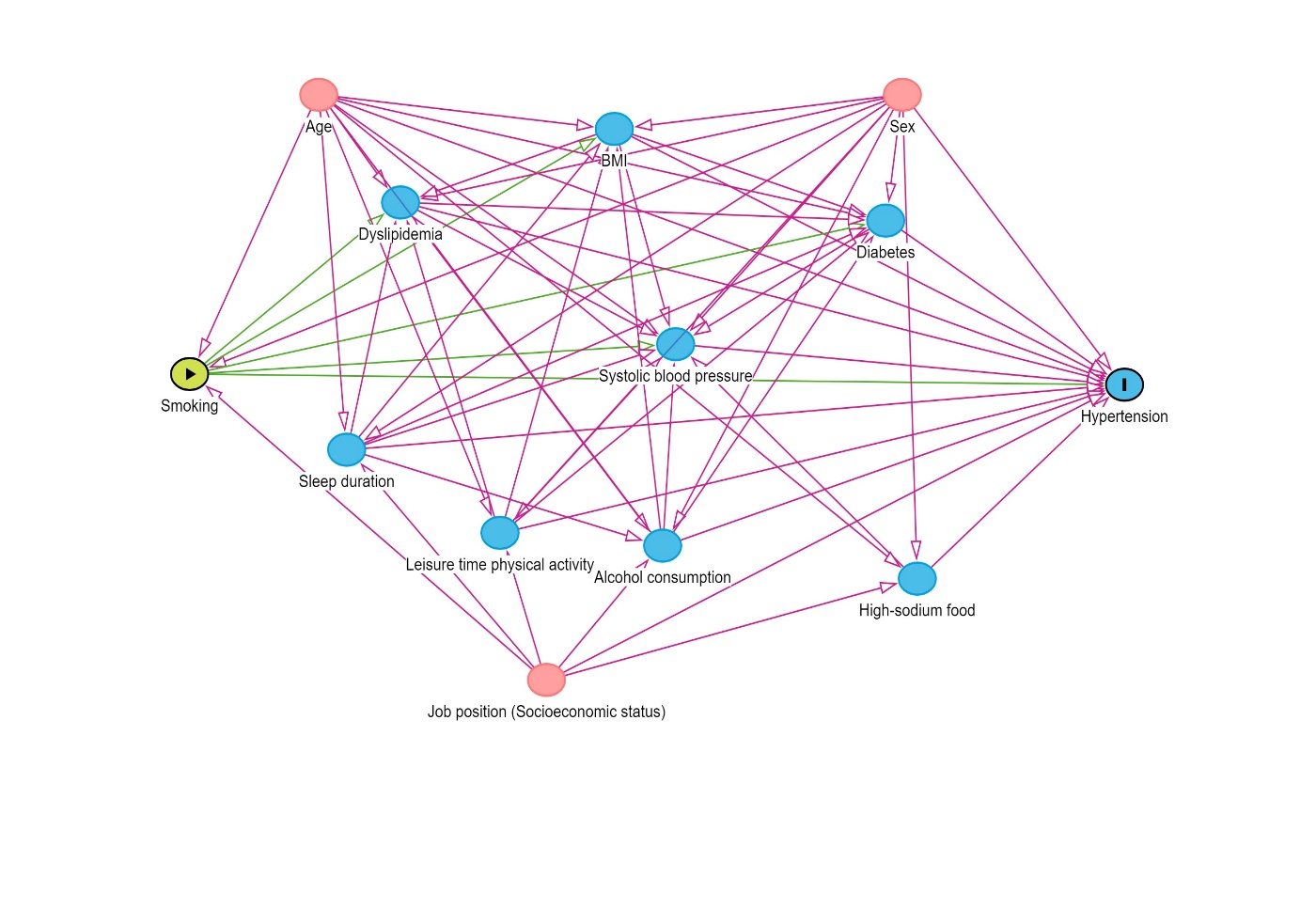


BMI, body mass index.

**Supplementary Figure S2 Participant health checkup attendance and dropout**

Year

Intermittent absence

Dropouts

Current participants

2018

30 152

25 041 (6.2%)

27 830 (5.6%)

29 603 (5.3%)

2020

2021

2019

549

456

1866

3245

Intermittent absence, people who did not attend the current health checkup but attended subsequent checkups. Percentage, the percentage of participants with missing data on smoking status.
